# The Book World of Medicine and Science

**Published:** 1896-05-23

**Authors:** 


### The Book World of Medicine and Science.

Uninhabitable and Insanitary Houses. By William Stevenson (a report of a lecture). (*Leith Express* Office, Leith. 1895.)

In this short lecture Mr. Stevenson, who from his position as a house factor may be imagined to have a practical acquaintance with his subject, enters into the causes which lead to houses becoming insanitary, and therefore uninhabitable, and the results of his investigations tend to support in the fullest manner the conclusion lately put forward in The Hospital that houses, and especially the houses of the working classes, should not be owned by their occupiers, but by large companies or syndicates. From the very moment they are erected houses require constant care and expenditure to keep them in repair, and this they do not get from people whose means are small. Especial attention is drawn to the evil results of leaving property in life-rent, which has been a fruitful cause of its rapid decay and ruin. A life-renter has no interest in maintaining property in any better repair than is sufficient to enable him to take as much out of the holding as possible. Needful repairs are executed with a grudge, and such improvements as would retain a good class of tenants are absolutely refused; the tenants, therefore, move, and their place is filled by a lower, and still lower class, and not only do the houses go to decay, but neighbouring properties become deteriorated past recovery. The same also happens when property is too heavily mortgaged.

Then the shortness of each individual life prevents that continuity of management which is essential for maintaining property in good sanitary condition.

The author would like to see house property taken up in large blocks by joint stock companies, and he sees no reason why a uniform and constant dividend of 4 to 4½ per cent. should not be made. The advantages and the economies of such a course would arise not merely from the continuity of the repairs, but especially from the fact that when whole and undivided blocks of houses are held the value of the property cannot be destroyed, as is so often the case now, by the carelessness of individual owners.

### DIRECTORIES.

We have received the following:—

Willing's British and Irish Press Guide. (London: J. Willing, 162, Piccadilly, W. Price 1s.)

This Guide to the press of the United Kingdom also contains a list of the principal English newspapers issued in the Colonies and British India, &c., and a descriptive list of papers in the English language printed on the Continent. It is so well arranged that references required are obtained instantaneously.

Mitchell's Newspaper Press Directory, 1896. (London: C. Mitchell and Co., 12 and 13, Red Lion Court, Fleet Street, E.C. Price 2s.)

As in previous years, this excellent Directory contains a carefully revised alphabetical index of the whole of the newspapers, magazines, and periodicals published in the United Kingdom, and a list of the Indian, colonial, and foreign press. A new feature introduced last year, viz., "The Bibliography of the Press," has been revised, added to, and brought up to date.

The City of London Directory for 1896. (London: W. H. and L. Collingridge, 148 and 149, Aldersgate Street, E.C. Price 12s. 6d.)

This useful work is invaluable to the man of business, and to anyone who requires information as to the location of City offices and buildings. A fitting complement to this Directory is the excellent coloured map which accompanies it.

